# Clinical Outcomes after Binocular Implantation of a New Trifocal Diffractive Intraocular Lens

**DOI:** 10.1155/2015/962891

**Published:** 2015-08-02

**Authors:** Florian T. A. Kretz, Detlev Breyer, Vasilios F. Diakonis, Karsten Klabe, Franziska Henke, Gerd U. Auffarth, Hakan Kaymak

**Affiliations:** ^1^International Vision Correction Research Centre (IVCRC) and David J Apple Laboratory for Ocular Pathology, Department of Ophthalmology, University of Heidelberg, 69120 Heidelberg, Germany; ^2^Breyer & Kaymak Augenchirurgie, 40212 Düsseldorf, Germany; ^3^Bascom Palmer Eye Institute, Miller School of Medicine, University of Miami, Miami, FL 33136, USA

## Abstract

*Purpose.* To evaluate visual, refractive, and contrast sensitivity outcomes, as well as the incidence of pseudophakic photic phenomena and patient satisfaction after bilateral diffractive trifocal intraocular lens (IOL) implantation.* Methods*. This prospective nonrandomized study included consecutive patients undergoing cataract surgery with bilateral implantation of a diffractive trifocal IOL (AT LISA tri 839MP, Carl Zeiss Meditec). Distance, intermediate, and near visual outcomes were evaluated as well as the defocus curve and the refractive outcomes 3 months after surgery. Photopic and mesopic contrast sensitivity, patient satisfaction, and halo perception were also evaluated.* Results.* Seventy-six eyes of 38 patients were included; 90% of eyes showed a spherical equivalent within ±0.50 diopters 3 months after surgery. All patients had a binocular uncorrected distance visual acuity of 0.00 LogMAR or better and a binocular uncorrected intermediate visual acuity of 0.10 LogMAR or better, 3 months after surgery. Furthermore, 85% of patients achieved a binocular uncorrected near visual acuity of 0.10 LogMAR or better.* Conclusions.* Trifocal diffractive IOL implantation seems to provide an effective restoration of visual function for far, intermediate, and near distances, providing high levels of visual quality and patient satisfaction.

## 1. Introduction

A new concept of intraocular lens (IOL) multifocality based on a refractive-diffractive technology has been recently developed, the trifocal IOLs. Multifocal IOLs based on this concept provide three useful focal distances, far, intermediate, and near, and therefore aim to provide functional visual restoration after cataract surgery [1–3]. These three foci can be generated by combining two bifocal diffractive profiles in one surface of the IOL [2] or by using a trifocal diffractive profile combined with a bifocal diffractive optic [1, 4]. Both manufacturing approaches for IOL trifocal technology have shown good visual and refractive outcomes that confirm the ability to provide efficient visual rehabilitation after cataract surgery [1, 4–8]. These outcomes have been confirmed to be consistent with the results of some optical simulations at the optical bench [9–11].

A new diffractive trifocal IOL was assessed in this study, AT LISA tri 839MP (Carl Zeiss Meditec, Jena, Germany), which combines a central 4.3 mm trifocal area with a bifocal diffractive surface between 4.3 and 6 mm of diameter [4, 5]. This IOL demonstrated in previous studies good visual, contrast sensitivity and optical quality outcomes [4, 5]. However, there is no study to date evaluating photic phenomena, such as glare or halos, which are relatively common with other types of multifocal IOLs [12]. Halos may cause a high level of dissatisfaction in spite of perfect refraction and above-average acuity values and can lead to the necessity of explanting the IOL [12]. Alba-Bueno et al. [13] published a study aimed at presenting the theoretical and experimental characterization of the halo in multifocal IOLs. These authors stated that the most noticeable characteristic of halos with the trifocal IOL AT LISA tri 839MP was the double-halo formation due to the 2 nonfocused powers. The aim of the current study is to evaluate the visual, refractive, and contrast sensitivity outcomes after binocular implantation of this trifocal IOL and also to evaluate the photic phenomena and the subjective satisfaction perceived by the patients. Therefore, this study tries to provide an integral analysis of the clinical outcomes obtained with this modality of trifocal IOL.

## 2. Methods

### 2.1. Population

This prospective nonrandomized study included consecutive patients scheduled to undergo bilateral cataract extraction for either visually significant cataract or refractive lens exchange, seeking for spectacle independence. In all cases, cataract surgery with bilateral implantation of the diffractive trifocal IOL AT LISA tri 839MP (Carl Zeiss Meditec, Jena, Germany) was performed. All patients were adequately informed and signed a consent form. The study adhered to the tenets of the Declaration of Helsinki and it was approved by the local ethics committee.

### 2.2. Inclusion and Exclusion Criteria

Patients were screened and included in the study only if they had an unremarkable ocular history and a preoperative corneal astigmatism of ≤1.25 D (diopters) (estimated postoperative corneal cylinder of ≤0.75 D based on the estimation of the surgically induced astigmatism by the clear corneal incisions). Patients with history of glaucoma or retinal detachment, corneal disease, irregular corneal astigmatism, abnormal iris, macular degeneration or retinopathy, neuroophthalmic disease, history of ocular inflammation, or previous ocular surgery were excluded from the study.

### 2.3. Preoperative and Postoperative Assessments

Before surgery, a complete ophthalmological examination was performed, including manifest refraction, keratometry (IOL Master v.4.3, Carl Zeiss Meditec, Jena, Germany), uncorrected (UDVA) and corrected (CDVA) distance visual acuity, Goldmann applanation tonometry, slit lamp examination, corneal topography (Pentacam HD, Oculus, Wetzlar, Germany), biometry (IOL Master v.4.3, Carl Zeiss Meditec, Jena, Germany), and funduscopy. Postoperatively, patients were examined the day after surgery as well as at 1 and 3 months after surgery. The postoperative examination protocol at 1 and 3 months was identical to the preoperative protocol, with the additional evaluation of monocular and binocular uncorrected (UIVA) and corrected (CIVA) intermediate visual acuity (66 cm), monocular and binocular uncorrected (UNVA) and corrected (CNVA) near visual acuity (40 cm) (all visual acuity measurements were performed under photopic conditions at 85 cd/m^2^), contrast sensitivity measurements under photopic (85 cd/m^2^) and mesopic conditions (3 cd/m^2^) with and without a glare source (Optec 3500 Vision Tester, Stereo Optical Co., using the Functional Acuity Contrast Test, FACT, Chicago, USA), determination of the best reading distance, and measurement of the binocular defocus curve to evaluate the range of functional vision. This curve was obtained with the patient wearing the correction providing the best distance visual acuity in both eyes, using ETDRS charts at a distance of 4 m, and introducing different levels of defocus in 0.5 diopter steps from +1.00 D to −4.00 diopters to identify the functional range of vision and the pseudoaccommodative range of a visual acuity of 0.3 LogMAR or better. In addition, patients were asked if they were satisfied with the results of the surgery (Yes/No) as well as if they perceived a disturbing level of glare or halos postoperatively (Yes/No). Likewise, patients were asked if they were satisfied in terms of the ability to read, intermediate and distance vision, quality of vision at all distances, and independence of spectacle for performing the daily activities and for the use of computer (Yes/No).

In all cases, the SRK/T formula was used for the calculation of the IOL power according to the measurements of corneal power, axial length, and anterior chamber depth obtained with the IOL Master system (Carl Zeiss Meditec, Jena, Germany). Target refraction was emmetropia in all cases.

### 2.4. Cataract Surgery Technique

Topical anaesthesia and mydriatic drops were instilled in all cases prior to the surgical procedure. All surgeries were performed by the same experienced surgeon (DB) using a standard technique of sutureless microcoaxial 2.2-mm phacoemulsification. All incisions were made at the steep axis of the cornea. After capsulorhexis creation and phacoemulsification, the IOLs were inserted into the capsular bag using the BLUEMIXS 180 injector (Carl Zeiss Meditec, Jena, Germany) through the main incision.

Postoperatively, all patients received the same treatment: a combination of an antibiotic, steroid, and nonsteroidal anti-inflammatory agent.

### 2.5. Statistical Analysis

SPSS statistics software package version 15.0.1 for Windows (IBM, Armonk, NY, USA) was used for statistical analysis. The Kolmogorov-Smirnov test was used to check the normality of the data distribution. When parametric analysis was possible, the Student *t*-test for paired data was performed for all parameter comparisons between preoperative and postoperative examinations as well as between consecutive postoperative visits. Otherwise, when parametric analysis was not possible, the Wilcoxon rank sum test was applied to assess the significance of differences between consecutive examinations. In all cases, the same level of significance (*p* < 0.05) was considered.

## 3. Results

The study enrolled a total of 76 eyes of 38 patients, 18 males and 20 females aged 62 ± 9.6 years (range, 34 to 79 years). Mean preoperative manifest sphere and cylinder were −0.02 D (range, −10.5 to +4.75 D) and −0.67 D (range, 0.00 to −1.75 D), respectively. Mean preoperative spherical equivalent was +0.21 D (range, −10.88 to +4.50 D) and mean preoperative LogMAR CDVA was 0.20 (range, 0.00 to 1.00). Mean IOL power implanted was 22.5 D, ranging from 15.50 to 25.50 D. Astigmatic locations were in 23% with the rule, 57% against the rule, and 20% oblique astigmatism.

### 3.1. Visual Acuity and Refractive Outcomes

**Figure 1:**
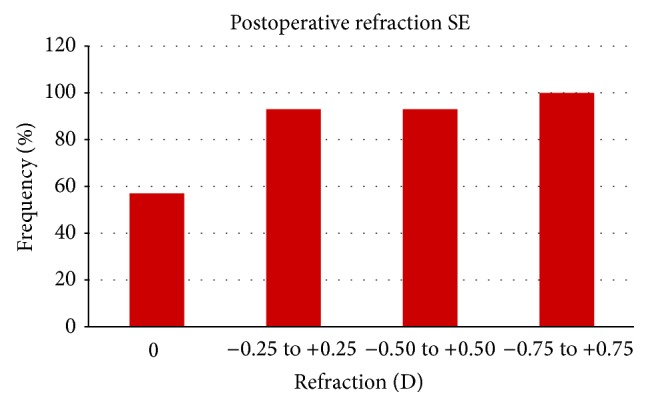
Distribution of the postoperative spherical equivalent (SE) at 3 months after surgery in the analysed sample.

**Figure 2:**
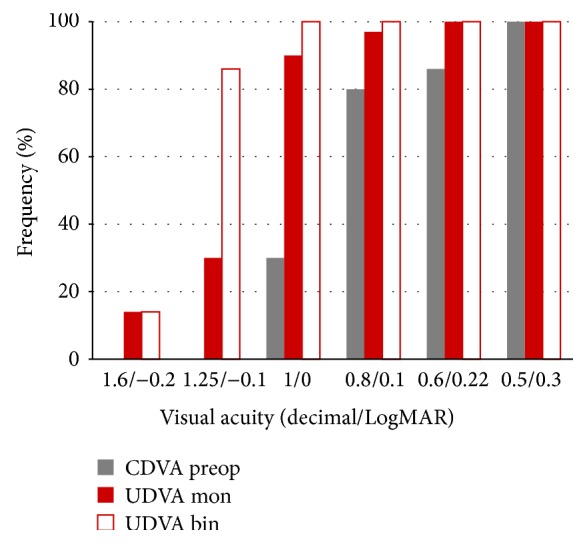
Postoperative distance visual outcomes at 3 months after surgery in the analysed sample. UDVA: uncorrected distance visual acuity; CDVA: corrected distance visual acuity; mon: monocular; bin: binocular; preop: preoperative.

Figure 1 shows the postoperative mean values of sphere (+0.05 D ±0.25), cylinder (−0.1 D ±0.18), and spherical equivalent (SE; −0.08 D ±0.25) as well as the distribution of the postoperative SE. A total of 90% and 100% of eyes had a postoperative SE within ±0.50 and 1.00 D of emmetropia. Mean monocular visual acuity was 0.10 for UDVA, 0.15 for UIVA, and 0.1 for UNVA, respectively. Mean binocular visual acuity was −0.05 for UDVA, 0.05 for UIVA, and 0.05 for UNVA. The distance visual outcomes are summarized in Figure 2. As shown, all patients had a binocular UDVA of 0.00 LogMAR or better, whereas 95% of eyes achieved this level of UDVA under monocular conditions (Figure 2).

**Figure 3:**
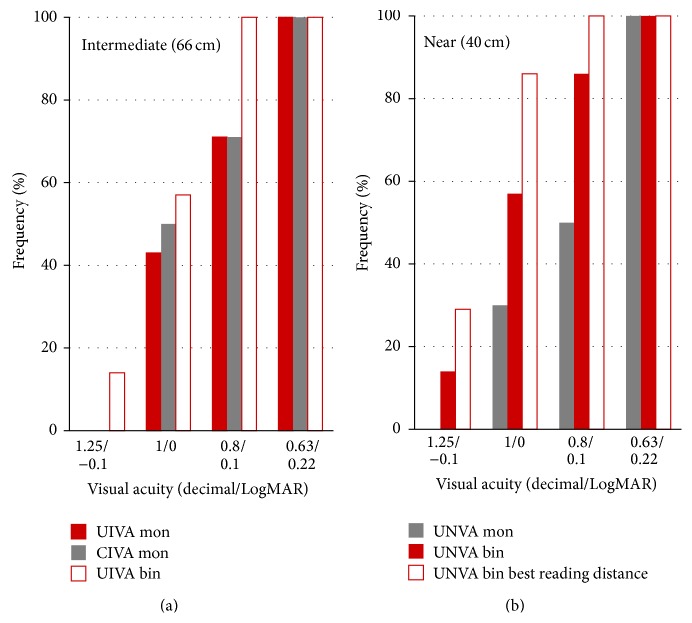
Postoperative intermediate (a) and near (b) visual outcomes at 3 months after surgery in the analysed sample.

Figure 3 displays the intermediate and near visual outcomes obtained in the analysed sample. As shown, all patients achieved a binocular UIVA of 0.10 LogMAR or better, whereas 70% of eyes achieved this level of UIVA monocularly. Likewise, 85% of patients achieved a binocular UNVA of 0.10 LogMAR or better in the analysed sample, and all eyes achieved a monocular UNVA of 0.20 LogMAR or better (Figure 3). Mean postoperative preferred reading distance was 38 cm, ranging from 32 to 40 cm. At this distance, all patients achieved an uncorrected binocular visual acuity of 0.1 LogMAR or better (Figure 3).

**Figure 4:**
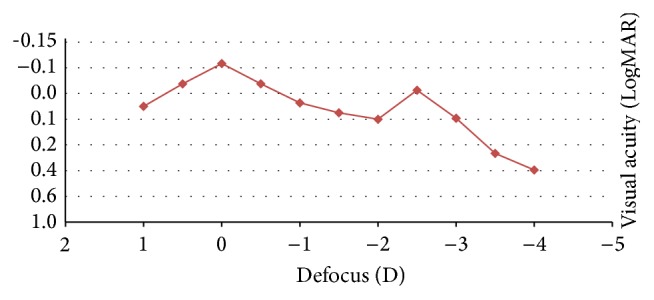
Mean binocular defocus curve at 3 months after surgery in the analysed sample.

Figure 4 displays the mean binocular defocus curve. As shown, acceptable levels of visual acuity were obtained, with the minimum value for a defocus of −2 D and the maximum value when no defocus was presented (Figure 4).

### 3.2. Contrast Sensitivity Outcomes

**Figure 5:**
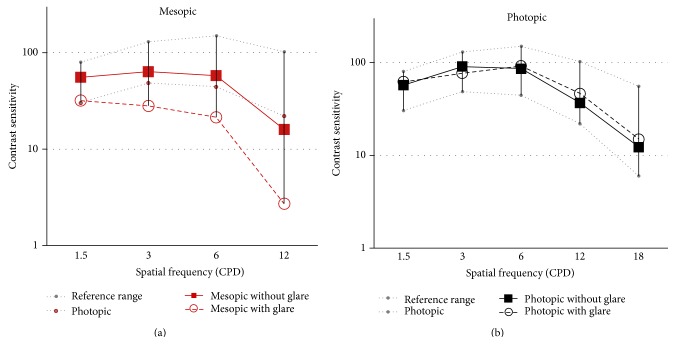
Mean contrast sensitivity function under mesopic (a) and photopic (b) conditions with (dashed line with circles) and without (straight line with squares) a glare source at 3 months after surgery in the analysed sample.

Mean contrast sensitivity function obtained in the group of eyes evaluated in the current study under photopic and mesopic conditions and with and without glare source is shown in Figure 5. As shown, photopic contrast sensitivity values measured with and without a glare source were not significantly different (*p* > 0.05) and were within the range of normality (Figure 5). In contrast, mesopic contrast sensitivity without a glare source was significantly higher than with glare (*p* < 0.05). Likewise, mesopic contrast sensitivity measured without glare was within the range of normality, except for the highest frequency evaluated, whereas mean values measured with glare were out of this range for all evaluated spatial frequencies (Figure 5).

### 3.3. Patient Satisfaction and Photic Phenomena

All patients (100%) were satisfied with the outcomes of surgery. Specifically, all patients (100%) were satisfied in terms of their ability to read, their intermediate and distance vision, their quality of vision at all distances, and their independence of spectacles for performing their daily activities and for the use of computer. Regarding the perception of photic phenomena, 90% of patients reported to perceive halos at 1 month after surgery, although 80% of these patients described these halos as not disturbing. At 3 months after surgery, the perception of halos decreased to 50%.

## 4. Discussion

In the current study, a good level of predictability has been found with the evaluated trifocal IOL, with 90% of eyes showing a SE within ±0.50 D. This confirms the refractive precision of the evaluated IOL, suggesting that the constant defined for IOL power calculation for the SRK/T formula is appropriate. Similar outcomes have been reported by other authors evaluating the same trifocal IOL [4, 5]. Law et al. [5] found in a prospective study a SE ranging from −0.50 to +0.75 D at all postoperative visits. Likewise, Mojzis et al. [4] found that 86.67% of eyes implanted with the same trifocal IOL had a postoperative SE within ±0.50 D.

The refractive precision in our study was consistent with the excellent distance visual outcome, with all patients and 95% of eyes achieving a binocular and monocular UDVA of 0.00 LogMAR or better, respectively. This distance visual outcome was consistent with that reported for the same trifocal IOL by other authors [4, 5] and equivalent to or even better than that reported for other types of trifocal IOLs [1, 6, 14]. Alió et al. [6] evaluated the visual outcomes of patients implanted with a trifocal IOL based on the combination of two bifocal diffractive patterns (FineVision from PhysIOL, Liege, Belgium) and found a mean postoperative monocular UDVA of 0.18 ± 0.13 LogMAR. In contrast, better visual outcomes and more similar to ours were reported by Cochener et al. [7] and Lesieur [8] with the FineVision IOL. Besides the optical performance of the trifocal IOL, several factors may have contributed to these differences between studies such as differences in the age of patients included in the sample, the sample size, nonoptimized IOL constants, or other methods of measuring visual acuity.

Regarding the near visual outcome, all eyes achieved a monocular UNVA of 0.20 LogMAR or better, and 85% of patients achieved a binocular UNVA of 0.10 LogMAR or better. This confirms the ability of the evaluated trifocal IOL to restore visual function at near distances after cataract surgery. It should be considered that the residual refractive error was almost zero in all cases (mean postoperative SE: +0.20 ± 0.30 D), with no eyes having a significantly myopic residual refraction biasing the measurement of UNVA. Our outcomes were better than those obtained by other authors evaluating the same trifocal IOL [4, 5]. Law et al. [5] found that binocular UNVA (40 cm) was 0.2 LogMAR or better in 77% of eyes and Mojzis et al. [4] obtained a mean value of monocular UNVA (33 cm) of 0.20 ± 0.12 LogMAR. This may be attributed to differences in the clinical protocol followed to measure the UNVA as well as to differences in the behaviour of the trifocal IOL within the eye due to differences in corneal power and axial length, and consequently in IOL power. In comparison to the trifocal IOL based on the combination of two bifocal diffractive patterns, our results were very similar to or even better than those reported by other authors [6–8]. Alió et al. [6] found a mean postoperative monocular UNVA of 0.26 ± 0.15 LogMAR (40 cm), whereas Cochener et al. [7] found a mean value of 0.01 ± 0.06 LogMAR (35 cm). As previously mentioned, besides differences in the optical performance of the IOL, other discrepancies in terms of clinical protocol and sample selection may have accounted for this variability between authors even for the same type of IOL.

Regarding intermediate vision, all patients achieved a binocular UIVA of 0.10 LogMAR or better, whereas 70% of eyes achieved this level of UIVA monocularly (measured at 66 cm). This also confirms the ability of the evaluated trifocal IOL to restore the intermediate visual function and is consistent with the outcomes reported by other authors evaluating the same type of IOL [4, 5]. Specifically, Mojzis et al. [4] reported a mean postoperative UIVA of 0.08 ± 0.10 LogMAR, also measured at 66 cm. Likewise, our results were consistent with those reported for the trifocal IOL combining two bifocal diffractive patterns [6–8] (FineVision IOL) but clearly better than those reported for another type of fully diffractive trifocal IOL (mean decimal UIVA of 0.58 ± 0.16 measured at 50 cm) [1]. Only the study of Alió et al. [6] reported a mean UIVA of 0.20 ± 0.11 LogMAR (40 cm) with the FineVision IOL, possibly due to differences in the clinical protocol followed or in the sample selection.

The defocus curve obtained for the evaluated trifocal IOL showed two clear peaks of maximum vision. This shape is similar to that obtained for the FineVision IOL by Alió et al. [6], Cochener et al. [7], and Lesieur [8] and somewhat different than that reported for the same trifocal IOL by Mojzis et al. [4]. The maximum visual acuity is achieved at distance, with a slight drop of visual acuity for defocus levels corresponding to intermediate vision and a slight visual recovery afterwards for defocus levels corresponding to near vision. A separate peak of acuity for intermediate vision was not expected due to the light distribution generated by the trifocal IOL evaluated, allocating less light to the intermediate focus (20%). In any case, for defocus levels between 0 and −3 D, a functional range of binocular visual acuity was maintained, with values of 0.1 LogMAR or better. In contrast, defocus curves of bifocal IOLs show a valley at the intermediate vision range.

The restoration of the distance, intermediate, and near visual function observed in the current series was accompanied by the achievement of a good contrast sensitivity outcome. Specifically, all postoperative contrast sensitivity values were within the range of normality, except for mesopic values obtained with a glare source. Similarly, Mojzis et al. [4] reported good photopic contrast sensitivity outcomes with the same type of trifocal IOL. Regarding the incidence of photic phenomena, halos were perceived in the initial postoperative period by a significant portion of patients, but most of them were reported as not disturbing. This perception of halos decreased in all patients at 3 months after surgery. Law et al. [5] also found a reduction in the perception of halos over time, decreasing from 80% at 1 month to 40% at 6 months after the implantation of the same trifocal IOL.

The visual function was restored after implantation of the evaluated trifocal IOL and most patients were satisfied with this implant. Although the light entering the eye is divided into three foci, a high level of corrected distance and near visual acuity as well as contrast sensitivity was achieved. This suggests that with this IOL enough light is allocated to each focus leading to providing a functional vision at far, intermediate, and near distances, with a controlled level of scattered light. Optical bench experiences have demonstrated that a trifocal IOL does not perform as well as monofocal or bifocal IOLs [10, 11], but possibly these differences in optical performance are filtered and neutralized by the neural processing. In terms of patient satisfaction, the ability of the trifocal IOL to provide a functional vision at all distances may overcome any relative limitation in optical quality.

Our study is limited due to small amount of eyes included; furthermore, reading speed is an important indicator of near visual performance and for this reason it is commonly included as an additional parameter in studies evaluating accommodating or multifocal IOLs. In the current series, reading speed was not evaluated which might be a limitation with regard to the assessment of functional vision. Furthermore, we did not use a validated quality of life questionnaire in this study; with our patient questionnaire we intended to assess the satisfaction of the patients with their visual performance and their ability to perform daily tasks and how much they are bothered by photic phenomena. Similarly, Law et al. [5] used a nonvalidated questionnaire to evaluate these same issues with the same modality of trifocal IOL, also finding high levels of satisfaction and low levels of visual disturbances due to glare or halos. Finally, one additional drawback of this study is to include both eyes of all patients in the analysis, as correlation of ocular data of fellow eyes of the same individual might be present. However, we decided to include both eyes of all individuals in order to avoid an additional reduction of the sample size and because binocular results are of high importance to assess the visual performance with this type of IOLs.

In conclusion, the trifocal diffractive IOL AT LISA tri 839MP is able to provide an effective distance, intermediate, and near visual restoration after cataract and refractive lens exchange surgery with high levels of visual quality and patient satisfaction and nondisturbing photic phenomena.
